# The United Kingdom Hospitals Conference in March

**Published:** 1908-01-25

**Authors:** 


### HOSPITAL ADMINISTRATION.

### CONSTRUCTION AND ECONOMICS.

### THE UNITED KINGDOM HOSPITALS CONFERENCE IN MARCH.

#### WANTED BUSINESS METHODS AND AN EXPERIENCED ADMINISTRATOR.

On December 6, 1906, at the instigation of the Joint Hospitals Committee of the British Medical Association, a Conference was held at University College, London, at which it was resolved to appoint a Committee in conjunction with the Joint Hospitals Committee of the British Medical Association and to arrange for a Conference in 1907. A good deal of interest was taken in the discussion, and there was an evident desire on the part of the Hospital representatives to get to work and do some business. It was believed also that the Joint Hospitals Committee of the British Medical Association was seriously wishful to infuse some life and activity into the administration of Hospitals, with a view to joint action and the introduction of modifications in certain directions. The necessity for business methods and active continuous work on the part of the Committee was made apparent at the Conference and afterwards, for without them the whole movement must be discredited and futile. Unfortunately a whole year has been allowed to elapse without any perceptible effort to bring the Committee together and to set to work on practical lines. Anyone who has read, or attempted to read, the Report published in the Journal of the meeting of the Joint Hospitals Committee on December 12 last must realise that it is hopeless to expect that any practical or useful purpose can be fulfilled until the organisation of a United Kingdom Hospitals Conference is taken out of the hands of the Joint Hospitals Committee of the British Medical Association and managed as a separate undertaking, with properly constituted Officers and a working Committee. As matters stand at present the organisation is without funds, it forms but a subsidiary portion of a mass of work undertaken by the British Medical Association, and no doubt for this reason the resolutions of the Conference of December 1906 have remained, to all intents and purposes, a dead letter, and the necessary steps to organise the proposed re-assembling of this Conference in March next have been left until now. If the hospitals are to subscribe money to defray the expenses of such a Conference, and to take any part or to display a genuine interest in it, it is essential that it should be promptly organised on independent lines, and that those responsible should exhibit business methods in the conduct of everything which appertains to its success. Otherwise it will be much better to face the position and admit that the movement initiated by the British Medical Association has failed and had better be allowed to drop altogether.

Mr. H. Manfield, M.P., and the Hon. Sydney Holland have consented to serve on the Sub-Committee which is charged with making all the necessary arrangements for the United Kingdom Hospitals Conference in March next. If this means, as we hope, that they will be given a free hand to organise the Conference on their own lines as men of business, independently of the British Medical Association, there is some hope that matters may yet take a practical form. The interval which has elapsed since the meeting in December 1906, and the publication of the Minutes of the Joint Hospitals Committee, prove to the hilt that at the present time the whole movement is hopelessly bound down by, and tied up in knots of tape of the very reddest hue. What is wanted is a Chairman who understands the subject, who will give time to the work, and who will put himself into it and stick to it and work at it. The absence of such a man has exhibited the intense weakness at the centre with regard to this movement, and it is necessary to point out that someone must decide what meetings are to be held and must have the courage to fix dates and summon meetings with regularity and despatch. As to the organisation of the work, it may be found advisable to have a separate Committee for the Scotch and Irish Hospitals respectively, but we doubt whether the Managers of the Scotch Hospitals would care to be dissociated from the English Hospital authorities. In any case the need of the moment is administration under the direction of a capable administrator. A knowledge of this fact led nearly everybody outside the British Medical Association to conclude, after the meeting of the last Conference, that the movement would end in nothing. If the Second Conference is to lead to any practical result it must be differently organised, and have for its motive a carefully thought out basis upon which future Conferences must rest.

#### Suggested Subjects for Discussion.

These are as follows:—

1. What provision should hospitals make with regard to cases of accident which are provided for under the Workmen's Compensation Act, 1906?
2. Methods of co-operation by hospitals with provident dispensaries and analogous institutions.
3. The operations of the "League of Mercy" as affecting the funds of provincial hospitals.
4. Whether the system of uniform accounts, as recommended by the King's Fund, should be used by all provincial hospitals.
5. Whether all hospital patients who can afford it should be compelled to pay for food and medicine supplied to them.
6. Pay wards.
7. The mode of election of honorary staffs of hospitals.

Of these, the first is of great practical value and should lead to some excellent results. It is remarkable, however, that the chief and most urgent matter, out-patient reform, by enactment of regulations providing that the out-patient department shall be made consultative in character, has disappeared. Does this mean that the Joint Hospitals Committee of the British Medical Association have come to the conclusion that they are not disposed to attempt to enforce the only system of control which is calculated to reduce hospital abuse in this department to a minimum, and to make the out-patient department in every hospital a real service to the public and the profession, as well as to the hospitals, instead of the very doubtful benefit which it is at present to any of the three?

It is not a little surprising, and shows a want of grasp and knowledge, that (3) the operations of the "League of Mercy" as affecting the funds of provincial hospitals, should find a place in this programme. The "League of Mercy" is rendering splendid service to provincial hospitals by extending the area of givers, and by giving directly in money to the support of provincial hospitals. Wherever it has started working, there the provincial hospitals have benefited in funds. It is not a little remarkable, therefore, that the Joint Hospitals Committee should have put upon the programme of the United Kingdom Hospitals Conference in March (3); for, as it is worded, it would seem to imply the exact contrary to the admitted and provable facts of the case.

We have advocated in The Hospital the co-operation of all British hospitals, the assembling of conferences, the interchange of information, the uniformity of account-keeping, and the general interchange of knowledge and goodwill among all these institutions throughout the Empire. For upwards of twenty-one years we have pursued this policy which has led to some remarkable results. We are, therefore, strongly in favour of the United Kingdom Hospitals Conference, but it must be the real thing, undertaken by the right people, and be placed in the hands of men who have proved their capacity to lead in the hospital world by the work they have done for the voluntary hospitals in various parts of the country in the past. We have felt it necessary to make this protest against any further playing with a great question, for the wasteful proceedings of the last fifteen months have brought great discredit upon the whole movement, and unless this grave neglect is finally ended or mended at the Conference in March, the whole proceedings had better be dropped.

